# The Association of *FLT3*-ITD Gene Mutation with Bone Marrow Blast Cell Count, CD34, Cyclin D1, Bcl-xL and hENT1 Expression in Acute Myeloid Leukemia Patients

**DOI:** 10.30699/ijp.2020.122579.2328

**Published:** 2020-07-16

**Authors:** Paulus Budiono Notopuro, Jusak Nugraha, Budi Utomo, Harianto Notopuro

**Affiliations:** 1 Faculty of Medicine, Airlangga University, Jawa, Indonesia; 2 Department of Clinical Pathology, Faculty of Medicine, Airlangga University, Jawa, Indonesia; 3 Department of Public Health, Faculty of Medicine, Airlangga University, Jawa, Indonesia; 4 Department of Biochemistry and Molecular Biology, Faculty of Medicine, Airlangga University, Jawa, Indonesia

**Keywords:** AML, Bcl-xL, Blast cell count, CD34, Cyclin D1, FLT3-ITD, hENT1

## Abstract

**Background & Objective:**

*FLT3*-ITD has been recently used as a molecular prognostic marker for risk classification in acute myeloid leukemia (AML) therapy. In this study we aimed to investigate the association of *FLT3*-ITD gene mutation with bone marrow blast cell count, CD34 expression as malignant cell burden, cyclin D1 and Bcl-xL expressions as indexes of cell proliferation and anti-apoptosis and human equilibrative nucleoside transporter 1 (hENT1) expression as cytarabine transporter during AML treatment.

**Methods:**

We investigated *FLT3*-ITD mutations, bone marrow blast cell count, CD34, cyclin D1, Bcl-xL and hENT1 expression in bone marrow aspirates from 22 de novo AML patients in a cross sectional study.

**Results:**

*FLT3*-ITD mutations were observed in 5 out of 22 de novo AML patients (22.7%). Patient with *FLT3*-ITD mutations had higher blast cell counts (79.5% vs 56.1%, *P*=0.004). In patients with *FLT3*-ITD mutations, CD34 and cyclin D1 expressions were higher (MFI 328.80 vs 25.78, *P*=0.003 and MFI 74.51 vs 57.15 *P*=0.005) than the patients without mutations. hENT1 expression in AML with *FLT3*-ITD mutation was lower (MFI 29.64 versus 56.32, *P*=0.0000) than in mutation-free AML. There was no significant difference in Bcl-xL expression between patients with and without mutations (*P*=0.61).

**Conclusion:**

A significant association was found between *FLT3*-ITD gene mutations in AML patients with bone marrow blast cell count, CD34, cyclin D1 and hENT1 expressions, however no association was obtained with Bcl-xL expression. These findings support the role of such mutation in pathogenesis of AMLand its contribution in rearrangement of standard therapy with cytarabine in management of AML.

## Introduction

Acute myeloid leukemia (AML) is a hematologic malignancy with clonal abnormalities in hematopoietic stem cells with heterogeneous clinical features and basic genetic aberrations. The incidence of AML is 4.2/100.000 persons/year globally and its mortality rate is 2.8/100.000 persons/year (1). Recently, it has been proposed that AML prognosis and risk stratification was determined based on the cytogenetic and molecular findings. The findings in favorable risk cases include inv16, t(16,16), t(8,21), t(15,17), normal cytogenetics with *CEBPA* biallelic gene mutation and *NPM1* gene mutation without *FLT3*-ITD mutation. Findings in intermediate risk cases include normal cytogenetics, +8 and t(9,11) and the findings in poor risk cases include complex cytogenetic abnormalities (>3 abnormalities), -5/5q-, -7, 7q- and 11q23 rearrangement except t(9,11), inv(3) or t(3,3), t(6,9), t(9,22) and normal cytogenetics with *FLT3*-ITD gene mutation (2).

There are various clinical pictures and different treatment outcomes in more than 50% of AML patients with normal cytogenetics (3). *FLT3*-ITD gene mutation has been reported to be a strong factor in newly diagnosed AML patients with normal cytogenetics and an independent prognostic factor that influences the treatment outcomes, overall survival and disease free survival. The frequency of *FLT3*-ITD gene mutation in AML patients is about 30% and varies around the world. Its frequency in de novo AML is higher than in secondary AML. *FLT3*-ITD gene mutation causes ligand independent dimerization and activation of FLT3 receptor. Autoactivation of FLT3 receptor leads to unregulated cell growth through excessive cell proliferation and anti-apoptotic activity (4,5). 

CD34+ cells are poor prognostic factors for complete remission achievement after induction therapy (6,7). Blast cell count and CD34+ cells reflect tumor burden and depend on cell proliferation and anti-apoptotic activity (8). *FLT3*-ITD gene mutation was reported to affect the human equilibrative nucleoside transporter 1 (hENT1) expression in children with acute lymphoblastic leukemia (9). hENT1 has an important role as a cytarabine transporter. Cytarabine is a major induction and consolidation backbone in AML treatment (10). 

In this study, we investigated the association of *FLT3*-ITD gene mutation with bone marrow blast count, CD34, Cyclin D1 (a cell proliferation marker), Bcl-xL (an anti-apoptotic agent) and hENT1 (a cytarabine transporter) expressions that lead to poor prognosis and determine treatment outcomes in AML patients with this mutation.

##  Materials and Methods

This study was a cross sectional study and was ethically approved by the ethical committee, faculty of medicine, Airlangga University. Appropriate informed consents were obtained before samples procurement. Bone marrow aspirates were collected from 22 newly diagnosed AML patients from August 2019 – January 2020 from a private hospital around Surabaya, Indonesia. All bone marrow samples investigated in the study were obtained at the time of first diagnosis. The laboratory examination was performed in department of biochemistry – molecular biology and clinical pathology, faculty of medicine, Airlangga University, Dr. Soetomo general hospital Surabaya.

Diagnosis of AML was established based on bone marrow aspirate cytology analysis. Diagnosis of AML was established observing more than 20% blast cell count as cut off point. Subtypes of AML were classified based on French American British (FAB) criteria. Bone marrow blast cells were counted among 500 of nucleated cells. The expressions of CD34, hENT1, Bcl-xL and cyclin D1 were examined with BD FACSCalibur^®^ using monoclonal antibody anti-CD45 conjugated to peridinin chlorophyll protein (PerCP), anti-CD34 conjugated to phycoerythrin (PE) from Becton Dickinson^®^, anti-hENT1 conjugated to fluorescein isothiocyanate (FITC), anti-Bcl-xL conjugated to PE and anti-cyclin D1 conjugated to FITC provided from Santa Cruz Biotechnology Inc^®^. 

The PCR investigation for *FLT3*-ITD gene mutation was done with bone marrow specimen. DNA was extracted and purified using QIAmp DNA blood mini kit Qiagen^®^. In brief, 1 µL of DNA was mixed with 10 µL of PCR kit Go Taq 2x PCR master mix solution (Promega^®^), 1 µL of forward and 1 µL of reverse primer in a total volume of 20 µL. The forward *FLT3*?????100 V electrophoresis was done with a 100 bp DNA ladder (Promega^®^). The presence of *FLT3*-ITD mutation was determined by visualization of 329 bp wild type gene fragment and a fragment larger than 329 bp. 

Of bone marrow aspirate with EDTA, 2 mL was examined for flowcytometry analysis. In brief, 50 µL of homogenous bone marrow aspirate and phosphate buffer saline (1:1) mixture was added to 2, 5 µL of PerCP-labeled anti-CD45, 2, 5 µL of PE-labeled anti-CD34 and FITC-labeled anti-hENT1 (pretreated with 1 mL of lysing solution, 250 µL of cytofix/cytoperm and 1 mL of perm wash reagent from Becton Dickinson^®^) in the first tube and 2, 5 µL of PerCP-labeled anti-CD45, 2, 5 µL of anti-Bcl-xL and 2, 5 µL of anti-cyclin D1 (pretreated with 1 mL of lysing solution, 250 µL of cytofix/cytoperm and 1 mL of perm wash reagent from Becton Dickinson^®^) in the second tube. Blast gating strategy was used to evaluate CD34, hENT1, Bcl-xL and cyclin D1 expressions in the blast cells. Median Fluorescent Intensity (MFI) was applied to examine CD34, hENT1, Bcl-xL and cyclin D1 expressions (11). 

**Statistical Analysis**

Independent sample t-test and Mann-Whitney U test were performed to compare quantitative data between AML patients with and without mutation. P-values less than 0.05 were considered statistically significant. All calculations were performed using the SPSS 22 (SPSS. Chicago, IL., USA). 

## Results

We analyzed bone marrow specimens obtained from 22 newly diagnosed AML patients for the presence of *FLT3*-ITD gene mutation, blast cell count, CD34, Bcl-xL, cyclin D1 and hENT1 expressions. Total AML patients included 13 males and 9 females. Both adult and pediatric AML cases (ages 4–84) were included in this study. According to FAB classification for AML, 6 cases were diagnosed with AML M1 (27.3%), 5 cases were diagnosed with AML M2 (22.7%), 5 cases were diagnosed with AML M3 (22.7%), 1 case was diagnosed with AML M4 (4.5%) and 5 cases were diagnosed with AML M5 (22.7%). 

**Fig. 1:**
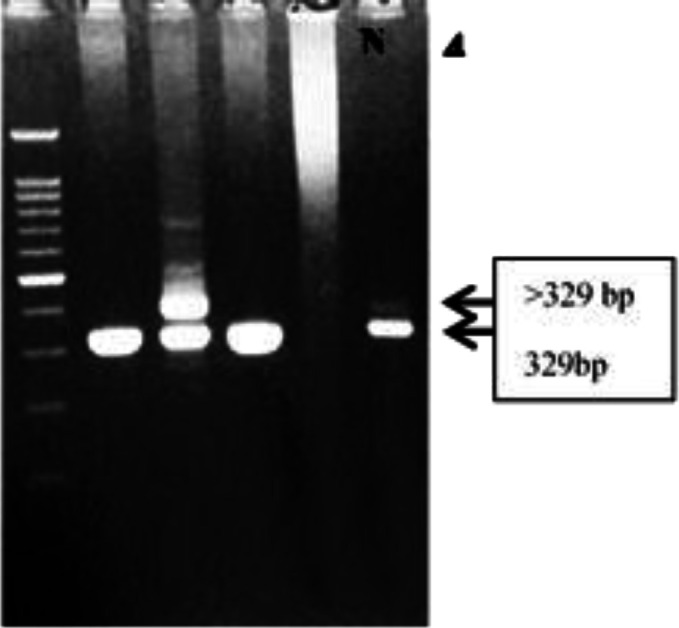
PCR results for detection of *FLT3*-ITD gene mutation. LD: DNA ladder 100 bp, NA: negative control, positive results of heterozygote mutant FLT3-ITD in patient number 2 (2 bands of 329 and > 329 bp), negative result (wild type) in patient number 1,3,4 (single band of 329 bp)

Mutations in *FLT3*-ITD were found in 5 (22.7%) AML patients based on the detectable amplicon with 329 bp and larger than 329 bp in 3% agarose gel electrophoresis. All of those patients were classified as *FLT3*-ITD mutants. The cases result of gel electrophoresis is presented in Figure 1.

**Table 1:** Values of blast cell count, expression of CD34, hENT1, cyclin D1, and Bcl-xL in AML patients with and without *FLT3*-ITD gene mutation

Parameter	AML with *FLT3*-ITD gene mutation (n = 5)	AML without *FLT3*-ITD gene mutation (n = 17)	P-value
Blast cell count - % mean + SD	79.5 + 12.9	56.1 + 14.2	0.004*
CD34+ expression (MFI) median	328.8	25.78	0.003*
hENT1 expression (MFI) mean + SD	29.64 + 5.10	56.32 + 12.81	0.0000*
Cyclin D1 expression (MFI) mean + SD	70.11 + 15.41	57.15 + 11.49	0.036*
Bcl-xL expression (MFI) median	208.28	188.7	0.61

* P-value <0.05

**Fig. 2.**
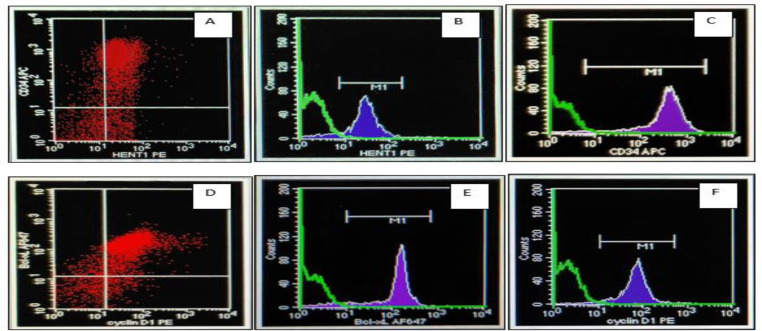
Flow cytometry analysis for the expression of CD34, Bcl-xL, cyclin D1, hENT1 in an AML patient with FLT3-ITD mutation. 2A and 2D) were the scattergrams for CD34, hENT1, Bcl-xL and cyclin D1 expression. 2B, 2C, 2E, 2F) were the histograms for CD34, hENT1, Bcl-xL and cyclin D1 expression (MFI analysis)

**Fig. 3:**
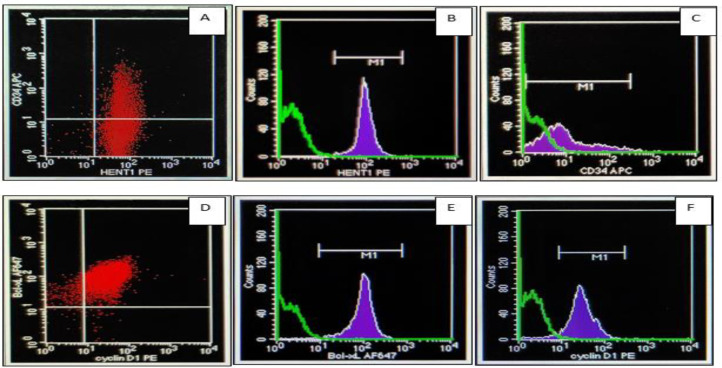
Flow cytometry analysis for the expression of CD34, Bcl-xL, cyclin D1, hENT1 in an AML patient without FLT3-ITD mutation. Figure 3A and 3D were the scattergrams for CD34, hENT1, Bcl-xL and cyclin D1 expression. Figure 3B, 3C, 3E, 3F were the histograms for CD34, hENT1, Bcl-xL and cyclin D1 expression (MFI analysis). Flow cytometry analysis for the expression of CD34, Bcl-xL, cyclin D1, hENT1 in an AML patient without FLT3-ITD mutation. Figure 3A and 3D were the scattergrams for CD34, hENT1, Bcl-xL and cyclin D1 expression. Figure 3B, 3C, 3E, 3F were the histograms for CD34, hENT1, Bcl-xL and cyclin D1 expression (MFI analysis)

The mutation of *FLT3*-ITD was frequently found in AML-M2 patients (3 out of 5 patients). The other subtypes of AML patients with *FLT3*-ITD mutations were AML-M3 (1 out of 5 patients) and AML-M5 (1 out of 5 patients). This mutation is found in 1 pediatric 5-year-old patient. The flowcytometry analysis of AML cases with and without FLT3-ITD gene mutation are presented in Figures 2 and 3.

The blast cell count in AML patients with *FLT3*-ITD gene mutation was significantly higher than mutation-free AML patients (79.5% vs 56.1%, *P*=0.004). The expressions of CD34 and cyclin D1 in AML cases with *FLT3*-ITD mutation were higher than in AML cases without this mutation (MFI 328.8 vs 25.78, p=0.003 and MFI 74.51 vs 57.15, *P*=0.005). hENT1 expression in AML with *FLT3*-ITD mutation was lower (MFI 29.64 vs 56.32, *P*=0.0000) than mutation-free AML. The Bcl-xL expression didn’t have a significant difference between AML patients with and without *FLT3* mutation (*P*=0.61). The analytical data are summarized in Table 1.

## Discussion

FLT3 is a tyrosine kinase receptor involved in hematopoiesis and commonly mutated in AML. There are two common mutations in *FLT3*. The first mutation is internal tandem duplication (ITD) in juxtamembrane (JM) domain and a point mutation in tyrosine kinase domain. *FLT3*-ITD gene mutation has been known as a strong prognostic factor in AML patients and it is related to disease progression, high relapse rate and low overall survival for 10 months (4,12,13). The frequency of *FLT3*-ITD gene mutation in AML is 21-24%. Its frequency is lower than *NPM1* gene mutation which is the most frequent mutation found in AML with the frequency of 35% in de novo AML and 45% in AML with normal cytogenetics. *FLT3*-ITD gene mutation in AML is definitively related to poor prognosis (5,14,15). 

In the current study, we determined the frequency of *FLT3*-ITD gene mutation and its association with blast cell count, CD34, cyclin D1, hENT1 and Bcl-xL expressions. The frequency of *FLT3*-ITD gene mutation in this study was 22.7% (5 out of total 22 AML patients). Our result is slightly higher than the published studies in Asian populations in which the frequency of *FLT3*-ITD gene mutation varies between 10 to 21% (13,16-18). The difference in mutation frequency could be due to differences in sample size, ethnicity, environmental factors and patient selection. The small sample size in this study does not represent the true frequency in population. In this study, the mutation is also more frequent in adult AML patients (80%) than in children, this result is similar to some previous studies (16,18).

Among various subtypes of AML based on FAB classification, in this study *FLT3*-ITD gene mutation is more frequent in AML-M2 patients (60%, 3 out of 5 AML patients). The other AML subtypes with *FLT3*-ITD gene mutation include AML-M3 (1 patient) and AML-M5 (1 patient). This result is different from the result of a large study in Germany. Thiede C. *et al.* reported that AML-M5 is the most common FAB subtype in AML with *FLT3*-ITD gene mutation (19). A study in a Chinese population showed that AML-M2 was the most frequent subtype in AML patients with *FLT3*-ITD gene mutations, while a study in a Thai population showed that AML-M3 was the most frequent subtype (16,17). The prognostic significance in AML-M3 patients with t(15,17) was still controversial (16,20).

Mutant FLT3 receptor inhibits the function of silencing mediator of retinoic acid and thyroid hormone receptor (SMRT), a co-repressor that interacts with promyelocytic leukemia zinc finger (PLZF) and eight twenty one protein (ETO) that are related to the cell proliferation blockade (4). Mutated receptor activates signal transducer and activator of transcription 5 (STAT5) that plays a critical role in cell proliferation and anti-apoptotic function. Leukemic cells harboring *FLT3*-ITD have high levels of STAT5 phosphorylation and increased bonding of this transcription factor to DNA. Activation of STAT5 plays a critical role in cell growth that is related to mitogen activated protein kinase (MAPK) and the regulation of cycline D1, Bcl-xL, c-MYC, PIM serine-threonine kinase and *p21* ^WAF1/CIP1^ (inhibitor of cyclin dependent kinase) transcription (4). 

*FLT3*-ITD mutation induces constitutive receptor activation, ligand independent dimerization and autophosphorylation which supports uncontrolled leukemic cells proliferation and apoptosis. The leukemic cells burden is the resultant of uncontrolled proliferation and apoptotic-antiapoptotic activity (4,21). In this study, we examined cyclin D1 as a cell proliferation marker and Bcl-xL as an anti-apoptosis activity marker of leukemic cells (22,23). AML patients with *FLT3*-ITD mutation had higher bone marrow blast cell count and CD34 expression level than mutation-free patients. Blast cell count and CD34 expression had significant association with the *FLT3*-ITD gene mutation in AML. Some studies have also reported that the presence of *FLT3*-ITD gene mutation is significantly associated with the higher blast cell count and CD34 expression (7,17,18,24). Normal *FLT3* and negative expression of CD34 predict a longer survival (25). 

In this study, cyclin D1 expression was significantly higher in AML with *FLT3*-ITD mutation than in patients without mutation. Cyclin D1 expression had a significant association with this mutation. This result supports the excessive leukemic cells proliferation in the pathogenesis of AML with *FLT3*-ITD mutation (4,22). *FLT3*-ITD gene mutation impairs the auto-inhibitory function in JM domain, causing ligand independent activation of the receptor and the receptor related pathway. The expression of Bcl-xL as the marker of anti-apoptotic activity was not significantly different in AML cases with and without *FLT3*-ITD gene mutations. The *FLT3*-ITD mutation was not associated with the expression of Bcl-xL. Based on this study, the excessive proliferation activity is the prominent pathogenesis of the AML with *FLT3*-ITD gene mutation.

AML patients with *FLT3*-ITD mutation have poor prognosis with high relapse rate, low overall survival (5,26). Our study showed that *FLT3*-ITD gene mutation had significant association with expression of hENT1, the important cytarabine transporter. AML patients with *FLT3*-ITD gene mutation had significantly lower expression level of hENT1 than mutation-free patients. Previous study proposed that *FLT3*-ITD mutation in AML cell lines had association with hENT1, an important cytarabine transporter in AML therapy (10). This finding suggested that *FLT3*-ITD specifically induced cytarabine resistance in leukemic cells through repression of hENT1 expression. Its mechanism might be due to upregulation of HIF-1α (10,27). Cytarabine is one of the standard chemotherapy drugs for AML (27,28). Our result supports the link between *FLT3*-ITD gene mutation and the resistance to cytarabine, one of the essential chemotherapy drugs in the current strategy of AML treatment in vivo. hENT1 is responsible for transporting cytarabine to the leukemic cells with up to 80% influx. Intracellular concentrations of cytarabine depend on the uptake process by hENT1 in AML induction treatment with standard doses of cytarabine (29-35). 

## Conclusion

*FLT3*-ITD gene mutation in AML patients was associated with blast cell count, CD34 and cyclin D1 expression, therefore it supports the role of *FLT3*-ITD gene mutation in excessive proliferation activity responsible for the pathogenesis of AML. This mutation was not associated with Bcl-xL expression.

*FLT3*-ITD gene mutation in AML is associated with hENT1 expression. The expression of hENT1 in AML patients with *FLT3*-ITD mutation is significantly lower than in mutation-free AML patients. This phenomenon supports the pathogenesis of cytarabine resistance in AML with mutated *FLT3*-ITD during AML induction therapy. It is important that either addition of FLT3 inhibitor in standard induction therapy and resetting cytarabine doses or development of novel therapeutic approaches should be considered in AML patients with *FLT3*-ITD gene mutation.
